# Epidermolysis Bullosa—A Kindler Syndrome Case Report and Short Literature Review

**DOI:** 10.3390/clinpract13040079

**Published:** 2023-07-30

**Authors:** Bogdan Ioan Stefanescu, Diana Sabina Radaschin, Geta Mitrea, Lucretia Anghel, Adrian Beznea, Georgiana Bianca Constantin, Alin Laurentiu Tatu

**Affiliations:** 1 Clinical Surgical Department, Faculty of Medicine and Pharmacy, “Dunarea de Jos” University, 800008 Galati, Romania; 2 Department of Obstetrics and Gynecology, Clinical Emergency Hospital “Sf. Ap. Andrei”, 800216 Galati, Romania; 3 Clinical Medical Department, Faculty of Medicine and Pharmacy, “Dunărea de Jos” University, 800008 Galați, Romania; 4 Dermatology Department, “Sf. Parascheva Clinical Hospital of Infectious Diseases”, 800179 Galati, Romania; 5 Neonatology Department, Clinical Emergency Hospital “Sf. Ap. Andrei”, 800216 Galati, Romania; 6 Morphological and Functional Sciences Department, Faculty of Medicine and Pharmacy, Dunarea de Jos University, 800008 Galati, Romania; 7 Research UDJ, 800008 Galati, Romania

**Keywords:** epidermolysis bullosa, genodermatoses, skin fragility, blistering disease, Kindler syndrome, prenatal testing

## Abstract

Introduction: Epidermolysis bullosa (EB) represents a group of rare disorders, genetically determined, characterized by skin fragility, blister formation and erosions due to minimal trauma. Depending on the ultrastructural level of skin cleavage, above or below the basement membrane, epidermolysis bullosa can be classified into four major types: simplex, junctional, dystrophic and Kindler Syndrome. In the junctional form of EB, the cleavage level is at the dermo-epidermal junction and the targeted proteins are laminin, type XVII collagen and integrins. The dystrophic form of EB is characterized by cleavage in the dermal layer, collagen VII being the targeted protein. In Kindler EB, multiple levels of cleavage have been described. The mutated gene is FERMT1. Another classification of this disease refers to phenotypic aspects such as extracutaneous lesions, severity, and distribution. The management of epidermolysis bullosa includes supportive wound treatments as well as nutritional support. Case report: We present a case of epidermolysis bullosa presented at birth, in a newborn with no family history of bullous skin conditions. The clinical presentation revealed extensive denuded areas and significant skin fragility as well as mucous and nail involvement. Prenatal diagnosis is very hard to achieve due to increased genetic heterogeneity of the disease. The short-term results were good. The importance of prenatal testing and possibilities of diagnosis are reviewed in this article. Conclusions: EB is a devastating disease. The presented case had a favorable evolution, with good short-term results. Significant morbidity can result from secondary infections of blisters and complications of the extracutaneous manifestations.

## 1. Introduction

Epidermolysis bullosa (EB) is a rare group of genodermatoses characterized by skin fragility and trauma-induced blister formation [1]. Based on the cleavage level, the classical forms of epidermolysis bullosa reveal four major types: simplex, junctional, dystrophic and Kindler Syndrome [2].

Epidermolysis bullosa simplex is the most common type, which only affects the outer layer of skin [1]. 

Junctional epidermolysis bullosa may be severe, with blisters beginning in infancy [1].

Dystrophic epidermolysis bullosa affects all the skin layers and it also involves mucous membranes, leading to extracutaneous manifestations [1].

Kindler syndrome is characterized by blistering in infancy, photosensitivity and progressive poikiloderma. It involves the skin and mucous membrane with radiological changes [1].

Bart syndrome is a rare condition characterized by epidermolysis bullosa (EB), aplasia cutis (AC), and nail abnormalities [2]. It is considered to be a clinical sign in many forms of EB [2].

The symptoms expressed vary from mild to severe depending on the histopathological presentation. Complications in the short term include malnutrition, dehydration, or sepsis. In the long term, epidermolysis bullosa is associated with high risk of squamous cell carcinoma development, contractures, or esophageal or laryngeal stenosis. The treatment is mainly supportive, focusing on wound and pain management [1]. 

The prevalence is not very well known. Mild cases are believed to occur with a frequency of 1 case per 50,000 births annually, whereas severe cases could be diagnosed in 1 case per 500,000 births annually [2]. 

It is genetically determined, usually autosomal dominant inherited, but there is a considerable genetic heterogeneity and recessive inheritance is observed in some variants [3]. Caused by mutations within the genes that encode keratins, desmosomes or hemidesmosomes or other intraepidermal or dermo-epidermal adhesion filaments, EB is characterized by poor cell adhesion, resulting in blister formation on different levels and lack of tissue repair or barrier function [4,5].

Prenatal genetic testing in high-risk families can be performed in very specialized centers. Although the disease has an impressive genetic heterogeneity, recent reports show encouraging data.

## 2. Case Report

We present the case of a 29-year-old pregnant woman, G2, P2, 39 weeks and 3 days of gestation, addressed to our department for uterine contractions. She had one previous pregnancy 5 years before, terminated at term by C-section due to acute fetal distress during labor, resulting in a live healthy female newborn weighing 2700 g, with an Apgar score of 8 at 1 min and 5 min.

There was no personal or familial history of congenital anomalies or genetic disease. The current pregnancy was uneventful; the patient was monitored from the first trimester. Ultrasound scans and blood samples for hematologic, metabolic or endocrinologic screening disorders were all within normal limits.

After taking the informed consent from the patient, and due to significant pain in the previous scar region, we decided to proceed with C-section. After consultation with a pediatrician and an anesthesiologist, the patient delivered, by C-section under spinal anesthesia, a live female neonate weighing 2800 g, with an Apgar score of 8 at 1 min and 5 min as well, with good adaptation. The placenta, umbilical cord and amniotic fluid were all macroscopically normal.

**Figure 1:**
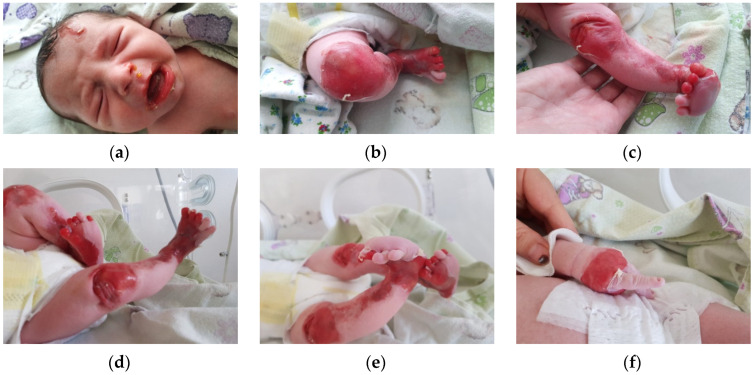
Clinical aspect at birth. (**a**) Well demarcated erosion and serous blister on forehead and periorificial erosions with mucous involvement; (**b**–**e**) Bart syndrome: extensive areas of denudation affecting the lower limbs, well demarcated red ulcerations and erosions, the surrounding cutaneous tissue is erythematous and reveals intense fragility, nail dystrophy and nail absence can be observed; (**f**) circumferential denudation of the hand.

The first clinical evaluation of the newborn revealed extensive areas of denudation, skin fragility and erosions. Blister formation on the frontal region of the face was observed, as well as denudation areas and periorificial involvement. The lower and upper limbs presented significant areas of denudation, multiple superficial erosions, and haemorrhagic blisters, as well as skin fragility. The clinical presentation of the lower limbs suggested Bart syndrome. The skin surrounding the lesions was erythematous and fragile. Nail dystrophy and nail absence were observed. No other anomalies were recorded (Figure 1a–f).

The newborn was transferred in the neonatal intensive therapy unit (NNITU). Under protection of intravenous vancomycin, a venous umbilical catheter was inserted and total parentheral alimentation was initiated: glucose 10%, aminoven 10%, calcium gluconate 10%. Skin lesions were cleaned with sterile 0.9% saline solution and ointment applied. 

Soft, loose-fitting clothes were used in order to avoid the appearance of new skin lesions and minimizing trauma.

Unfortunately, the dermatological status of the newborn worsened in terms of the new appearance of bullae leaving large areas of denuded skin, and it was decided to transfer the case to a higher specialized center for further care. The Birmingham Epidermolysis Bullosa Severity score (BEBS) on admission was 10.5. During hospitalization, the newborn received prophylactic antibiotic therapy with vancomycin 15 mg/kg per day for 10 days, ceftazidime 20 mg/kg per day for 5 days and meropenem 20 mg/kg per day for 5 days, as well as fluconazole to prevent mycosis. Gavage enteral feeding was initiated on day 2 and was associated with partial parenteral alimentation for the next 10 days. 

Skin and mucosal lesions were carefully treated using Cicaplast soothing cream, Grassolind ointment compresses which are permeable to air and exudate and less traumatic Peha Crepp and Tubifast bandages.

**Figure 2:**
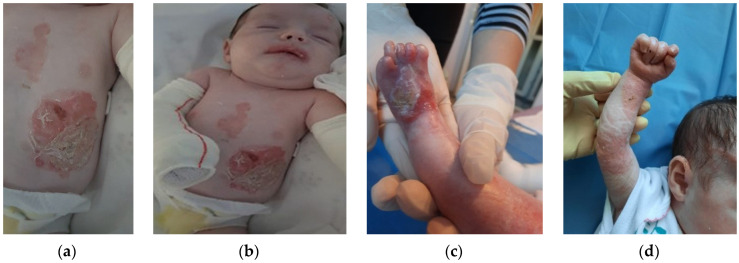
Clinical presentation at 5 months of age. (**a**) Widespread nonhealing erosions, crusting and scarring on the trunk; (**b**) Scarring and erosions affecting the lips; (**c**) Nonhealing ulcerations, crusts and atrophic scars on the left foot, nail loss and pseudosyndactyly; (**d**) atrophic scars, millia and nail dystrophy on the right arm.

**Figure 3:**
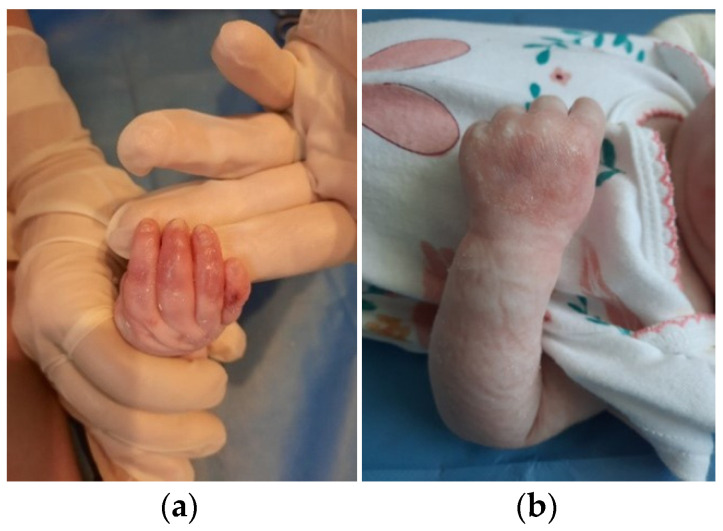
Clinical presentation at 6 months of age. (**a**) Nonhealing erosions, atrophic scarring and nail dystrophy on the right arm; (**b**) Atrophic scars and skin fragility on the left arm.

**Figure 4:**
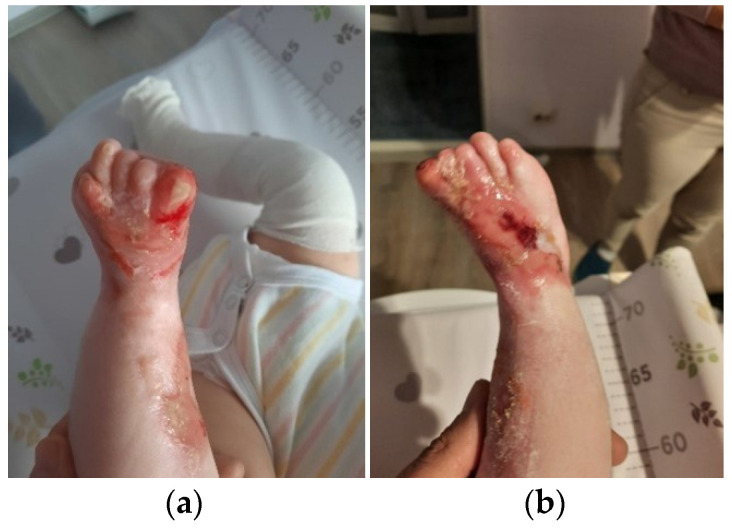
Clinical presentation at 8 months of age. (**a**) Chronic ulcerations, crusting and atrophic scars affecting the left foot, nail dystrophy, nail loss and pseudodactyly; (**b**) Extensive denudation area, crusts and scarring on the right foot, nail dystrophy, nail loss and pseudodactyly.

The patient was discharged after 41 days of hospitalization. Long-term follow-up of the baby revealed the appearance of new lesions and vicious cicatrization of the previous ones, leading to syndactyly at the age of 8 months (Figure 2a–d, Figure 3a,b and Figure 4a,b).

Moreover, due to alimentation difficulties, the weight gain of the baby was below normal values. At 9 months, the patient weighed only 7.2 kg, 7th percentile, and the alimentation was mainly liquid or semiliquid, bottling. The physical development of the baby was unsatisfactory. Due to pain, restrictive dressings and skin lesions, the motor skills of the infant were limited. Sitting with support was only possible at the age of 9 months. 

Genetic tests for both parents and baby were recommended. Mutations in the COL7A1 gene, which encodes type VII collagen, were identified. The paternal karyotype showed mutation of the c.7547dup gene whereas maternal karyotype revealed the mutation of the c.6788G>T gene. The molecular karyotype of the baby showed multiple gene mutations such as c.7547dup and c.6788G>T, associated with alterations in type VII collagen. Mutations in gene COL7A1 are highly suggestive in DEB (Dystrophic Epidermolysis Bullosa). COL7A1 encodes the type VII collagen, the dominant constituent of the anchoring filaments of the epidermal-dermal junction. DEB can be inherited either in an autosomal dominant or autosomal recessive manner. The types of mutations observed in COL7A1 could account for the genetic heterogenicity expressed in DEB. Therefore, the mutations observed in our case could explain the possible pathogenic implications to only one child in the family, the older sibling not showing any clinical sign of the disease. The karyotype of the infant also showed mutations in the FERMT1 gene. Mutations in the FERMT1 gene are usually associated with Kindler syndrome.

The postpartum course of the mother was uneventful. She was discharged at the 4^th^ postoperative day. 

Parents were counseled regarding the overall prognosis and instructed in nursing and wound management.

## 3. Discussion

The incidence and prevalence in the USA are estimated at approximately 19 cases per 1 million live births and 11 cases per 1 million population, respectively [6,7]. Similar numbers are reported in Europe, Asia and Australia [8,9,10].

More than 10 eponyms have been used to designate different forms of the disease, such as Dowling-Meara, Weber-Cockayne, Kallin, Mendes de Costa, Herlitz and so on. After an international consensus meeting held in 2008 in Vienna, Austria, four major types of EB were proposed [11].

Depending on the level of blister formation, EB is actually classified in four major types: EB simplex (EBS), in which the blister develops intraepidermally; junctional EB (JEB), where the defect is observed at the junctional level, in the lamina lucida; dystrophic EB (DEB), where the blistering occurs below the basement membrane, within the lamina densa; and Kindler Syndrome (KEB), with various split levels [12].

In EBS, blister formation is intraepidermal within the keratinocytes of the basal layer. It is the most frequent form of EB. The identified genes responsible for this form of EB are those that encode keratins 5 and 14. Clinically, EBS encompasses trauma-induced lesions with blistering occurring primarily on the hands and soles, healing without scars, with restitutio ad integrum in mild cases. Severe cases have been reported with extracutaneous lesions and generalized blistering, resulting in a poor outcome due to sepsis or feeding inability [12,13]. 

In JEB, the blister formation is observed in the lamina lucida or in the superior part of the lamina densa. The blistering affects both the skin and mucosae and heals with scarring. Some variants of JEB can affect internal organs, leading to airway obstruction due to scarring or blister formation. In the severe form (Herlitz JEB), mild blistering is present at birth but generalized blistering rapidly occurs, leading to a poor outcome. Pathognomonic for this variant are the plaques of exuberant granulation affecting the oral cavity, face or nose [12,14,15].

The mutations observed in DEB refer to the genes that encode collagen type VII, which represents a key element in the anchoring of the epidermis to the dermis. The cleavage is observed below the lamina densa. Collagen VII is present not only in the skin but also in the mucosae and the upper esophagus, so that blistering can affect the ocular, nasal, oral and anal mucosae as well as the upper third of the esophagus. DEB can be inherited in an autosomal dominant or recessive manner, but phenotypic overlap may be possible. In the most recent classification, dated 2020, DEB is subdivided into 4 major types [16]:

1. Localized dominant dystrophic EB;
2. Intermediate dominant dystrophic EB (previously known as generalized DDEB);
3. Intermediate recessive dystrophic EB (previously known as non-Hallopeau–Siemens RDEB);
4. Severe RDEB (Previously known as Hallopeau–Siemens RDEB).

DEB manifests with skin fragility and blistering but the severity of the lesions depends on the clinical variant of the disease. The most severe type of DEB, previously known as Hallopeau–Siemens syndrome, can present with extensive denudation at birth, pseudosyndactyly due to scarring, contractures of the hands and feet and mucosal involvement which can have mutilating effects. The teeth are dystrophic and mouth restriction may be observed due to severe scarring, resulting in food deprivation. The esophagus may present strictures and scarring alopecia is a frequent finding on these patients. 

The dominant forms of DEB appear to have a better prognosis, lacking the mutilations observed in the recessive variants. In dominant dystrophic EB, the mucosae may be spared, and blistering may not be so extensive. 

The risk of developing squamous cell carcinomas (SCC) is high in all four variants of EB, and close monitoring should be performed. SCC develops on chronic wounds in adults, and is characterized by an aggressive course with frequent recurrences after surgical treatment [17].

Kindler syndrome, or congenital bullous poikiloderma, has an autosomal recessive inheritance and is characterized by trauma-induced blistering on acral skin, which heals with atrophic scars. Also, there is a degree of photosensitivity, which may decrease with age, and poikiloderma on sun-exposed areas. Other symptoms include palmo-plantar hyperkeratosis and pseudosyndactyly. Mucous membranes can be affected, leading to loss of teeth, ectropion, esophageal strictures or phimosis. Blistering occurs at multiple levels in the same individual, so the histopathological examination can reveal cleavage in the dermis or the junctional membrane [12,18].

Bart syndrome represents a rare, severe clinical manifestation observed in different subtypes of epidermolysis bullosa [19]. The disorder was reported by Bart et al. in 1966 highlighting three features: congenital skin denudation, blister formation and nail anomalies [20].

The certitude diagnosis for EB is achieved using skin biopsies for electron microscopy of specific lesions from EB, such as those inducing the formation of new bullae. Electron microscopy objectifies the level of cleavage and the subtype of the disease. Another method in the diagnostic procedure of EB is immunofluorescence microscopy. Immunohistochemistry can be performed with keratin 14 or collagen IV, and thus determines the level of cleavage and the molecular defect. Immunofluorescence antigen mapping uses specific antibodies directed against different proteins localized at the epidermal-dermal junction in order to identify the type and subtype of the disease. The antibodies used are mainly against collagen VII, IV, XVIII and laminin [21]. In EB simplex, the proteins targeted are represented by the plakin group (desmoplakin, plakoglobin, plakophilin), or keratin 5–14, plectin and exophilin-5. In junctional EB, collagen XVII and laminin (LAMA3, LAMB3, laminin 332) are the proteins targeted. In dystrophic EB, collagen VII is the protein targeted [22]. 

Prenatal diagnosis is very hard to achieve due to increased genetic heterogeneity of the disease. Identified mutations in more than 10 structural genes expressed in the cutaneous basement membrane zone reported so far, along with a better genotype–phenotype correlation, could have a tremendous positive effect on prenatal diagnosis [20]. Genetic testing of the fetus in high-risk families—chorionic villus sampling at 12 weeks of gestation or amniotic fluid samples at 16 weeks of gestation—allows the prediction of the disease status as well as the prevention of the birth of a severely affected child [22]. Moreover, recent advances in non-invasive genetic testing, such as cell-free fetal DNA, could also be applied in the detection of EB.

On the other hand, there are reports in the literature about prenatal diagnosis of EB successfully established using fetal skin biopsies. Considering all the risks associated with this procedure, this technique cannot be used as a screening method and should only be used in high-risk families. Isolated cases of EB with no familial history of blistering disease, such as our case, could not benefit.

Complications of EB derive from the muco-cutaneous lesions. Sepsis is due to bacterial colonization of the widespread ulcerations. Because of the oral and esophageal lesions, the patient is incapable of feeding and malnutrition is observed. There is a high risk for airway obstruction when the larynx or trachea are affected by blisters. Phimosis, urethral strictures or even nephrotic syndrome may occur. The risk of SCC in patients suffering from EB, especially RDEB, is very high and close monitoring is necessary.

There is no specific treatment for EB. Wound care and prevention of bacterial colonization are essential in the management of EB. The management of EB implies the collaboration of multidisciplinary teams. A multidisciplinary approach is important for prevention and treatment of complications that occur during the course of the disease [23]. 

Good wound care is extremely important. The use of topical and systemic antibiotics is essential for infected lesions and ulcerations in order to reduce the morbidity. Moisturizers are recommended (to reduce xerosis and skin fissures), together with protection from trauma and the sun [24]. Screening for premalignant keratoses should begin in adolescence [16]. The treatment administered in this case is similar to what we found in the literature. Different types of foams, modified absorbent pads, lipidocolloid dressings, contact layers, hydrogel sheets and alginates can be used. The fact that there are so many wound care products can complicate the decision, and there are no specific guidelines [24]. The clinical variability requires an individualized treatment.

Another important aspect is pain management. Debridement of the lesions is necessary for older lesions. Different nociceptives, such as acetaminophen or even opioids [25], can be used. The patient must be advised to prevent minimal trauma and to use sunscreen protection as often as possible, given the risk of carcinomas.

## 4. Conclusions

EB is a devastating disease. Patients should modify, adapt and limit their activity in order to avoid blistering and subsequent complications, or use appropriate cushioning of sites of friction in order to avoid trauma to the skin. The prognosis is, in most cases, poor. Death may occur during infancy or early childhood, mainly due to sepsis, renal failure or upper airway obstruction.

The presented case had a favorable evolution, with good short-term results. It is essential to advise the family to avoid trauma, in order to prevent blister formation. Patients may have a normal life, but significant morbidity can result from secondary infections of blisters and complications of the extracutaneous manifestations.

In the future, prenatal screening using non-invasive genetic testing should be developed and used, at least for early diagnosis of a severely affected fetus. 

## Data Availability

No new data were created or analyzed in this study. Data sharing is not applicable to this article.
